# Malaria surveillance-response strategies in different transmission zones of the People's Republic of China: preparing for climate change

**DOI:** 10.1186/1475-2875-11-426

**Published:** 2012-12-21

**Authors:** Guo-Jing Yang, Marcel Tanner, Jürg Utzinger, John B Malone, Robert Bergquist, Emily YY Chan, Qi Gao, Xiao-Nong Zhou

**Affiliations:** 1 Jiangsu Institute of Parasitic Diseases, Wuxi, Jiangsu, 214064, People’s Republic of China; 2 Key Laboratory on Control Technology for Parasitic Diseases, Ministry of Health, Wuxi, Jiangsu, 214064, People’s Republic of China; 3 School of Public Health and Primary Care, The Jockey Club Chinese University of Hong Kong, Shatin, Hong Kong; 4 Department of Epidemiology and Public Health, Swiss Tropical and Public Health Institute, P.O. Box, CH-4002, Basel, Switzerland; 5 University of Basel, P.O. Box, CH-4003, Basel, Switzerland; 6 Pathobiological Sciences, Skip Bertman Drive, Louisiana State University, Baton Rouge, LA, 70803, USA; 7 , Ingerod, Brastad, Sweden; 8 National Institute of Parasitic Diseases, Chinese Center for Disease Control and Prevention, Shanghai, 200025, People’s Republic of China; 9 Key Laboratory on Biology of Parasite and Vector, Ministry of Health, People’s Republic of China, WHO Collaborating Center for Malaria, Schistosomiasis and Filariasis, Shanghai, 200025, People’s Republic of China

**Keywords:** Malaria, Climate change, Surveillance-response, Elimination, People’s Republic of China

## Abstract

**Background:**

A sound understanding of malaria transmission patterns in the People’s Republic of China (P.R. China) is crucial for designing effective surveillance-response strategies that can guide the national malaria elimination programme (NMEP). Using an established biology-driven model, it is expected that one may design and refine appropriate surveillance-response strategies for different transmission zones, which, in turn, assist the NMEP in the ongoing implementation period (2010–2020) and, potentially, in the post-elimination stage (2020–2050).

**Methods:**

Environmental data obtained from 676 locations across P.R. China, such as monthly temperature and yearly relative humidity (YRH), for the period 1961–2000 were prepared. Smoothed surface maps of the number of months suitable for parasite survival derived from monthly mean temperature and YRH were generated. For each decade, the final malaria prediction map was overlaid by two masked maps, one showing the number of months suitable for parasite survival and the other the length of YRH map in excess of 60%.

**Results:**

Considering multiple environmental factors simultaneously, the environmental variables suitable for malaria transmission were found to have shifted northwards, which was especially pronounced in northern P.R. China. The unstable suitable regions (transmission periods between five and six months) showed increased transmission intensity due to prolonged suitable periods, especially in the central part of the country.

**Conclusion:**

Adequate and effective surveillance-response strategies for NMEP should be designed to achieve the goal of malaria elimination in P.R. China by 2020, especially in the zones predicted to be the most vulnerable for climate change.

## Background

Malaria remains an infectious disease of foremost public health importance in many parts of the world, including the People’s Republic of China (P.R. China) [1]. Historically, high malaria incidence rates have been reported from 24 provinces of P.R. China [2]. Owing to large-scale control activities facilitated through primary healthcare networks and community participation, malaria due to *Plasmodium falciparum* has been eliminated in most provinces, except Yunnan and Hainan [3-6]. Meanwhile, *P. vivax* has become the predominant species. Although this species causes less morbidity and mortality than that of *P. falciparum*, the persistence of *P. vivax* through dormant stages (i.e., hypnozoites), is a major impediment toward complete elimination of malaria in P.R. China [7-9]. Moreover, the country is vulnerable to imported malaria [5,10,11], due to intense population movements within the country [12,13], and influx from neighbouring countries, such as Lao People's Democratic Republic (Lao PDR), Myanmar, and Vietnam [10].

Malaria transmission is governed by social-ecological systems. Previous studies carried by Yang et al. [14] suggest that temperature and relative humidity (RH) are the most important determinants for malaria transmission among all investigated environmental factors. Laboratory experiments have determined that *P. vivax* development ceases within the *Anopheles* spp. mosquito at 14.9°C [15,16]. Usually, temperatures below 16°C and over 30°C are not compatible with the survival of the a vector and could thus restrain the development of the *Plasmodium* sporocysts in the mosquitoes [17]. Although the RH shows no direct effect on the development of *Plasmodium* spp., it exerts an influence on transmission through the activity and life-span of the vector, i.e., the lower the RH, the shorter the life-span of the vector [18]. For instance, malaria transmission is strongly inhibited at a yearly RH average below 60% [19,20]. Climate change might well exacerbate the effect of environmental determinants on malaria transmission, particularly rising temperature. Indeed, within the survivable range of temperatures, mosquitoes proliferate faster and show higher biting rates as temperature increases [21]. Moreover, warmer conditions speed up the development of pathogens within their hosts. For example, at 19°C, it takes 30 days for an immature *P. vivax* to develop fully within the vector, while only 13 days are required at of 28°C [16].

Mathematical models, coupled with geographical information system (GIS), revealed an increase of the population at risk of malaria transmission due to climate change [22,23]. For example, an overall warming of 0.5°C in the East African highlands is predicted to result in a 30-100% increase in mosquito abundance [24]. However, other studies failed to demonstrate a clear link between climate change and an elevated risk of malaria [22,25], underscoring the complexity and high levels of uncertainty in current predictions [26,27]. New research is therefore needed to investigate how environmental factors affect the transmission of malaria in different parts of the world. In 2010, P.R. China launched its national malaria elimination programme (NMEP). Hence, it is important to deepen the understanding of malaria transmission patterns, which might help to design more effective surveillance and response strategies for the NMEP [28].

Here, the spatial and temporal patterns of *P. vivax* transmission in P.R. China are predicted, using an established biology-driven model. The predicted patterns, in turn, will guide the design and refinement of appropriate surveillance-response strategies for different transmission zones, and thus assist in optimizing the NMEP in the current ongoing implementation period (2010–2020) and, potentially, in the post-elimination stage (2020–2050).

## Methods

### Environmental data

The digital map database of P.R. China (DMDC), available at a scale of 1:1,000,000 (National Bureau of Surveying & Mapping, P.R. China), was used to delineate administrative boundaries for subsequent disease mapping. With regard to environmental data, the average daily temperature and RH were obtained from 676 locations evenly distributed across P.R. China. These environmental data cover a 40-year time series (1961–2000), and are provided by the Chinese Meteorological Administration (http://www.cma.gov.cn). In order to observe the evolvement over longer periods, the monthly/yearly environmental data for each decade were determined as detailed below.

For each decade, the monthly mean temperature and RH were calculated according to equations (1) and (2):

(1) $$\text{Temperatur}e_{\text{month}}=\frac{\sum_{1}^{10}\left(\left(\sum_{1}^{1}T_{\text{daily}}\right)/i\right)}{10}$$

(2) $$\text{Temperatur}e_{\text{month}}=\frac{\sum_{1}^{10}\left(\left(\sum_{1}^{1}RH_{\text{daily}}\right)/i\right)}{10}$$

where *T*_*daily*_ and *RH*_*daily*_ signify the daily mean temperature and RH, respectively, whereas *i* denotes the number of days in a month.

Since the lowest temperature required for the development of *P. vivax* within the *Anopheles* vectors is 14.9°C, the number of months suitable for parasite survival over the year (*N*_*month*_) was calculated for each location. There is no region of their monthly average temperature higher than 30°C which is the highest thermal threshold for development of *P. vivax*.

According to historical records, the vector of *P. vivax* occurs primarily in regions where the yearly average RH (YRH) exceeds 60% [14]. It is important to take this factor into account when determining the vector distribution. The YRH is calculated according to equation (3):

(3) $$\text{YRH}=\frac{\sum_{1}^{10}\left(\left(\sum_{1}^{365}RH_{\text{daily}}\right)/365\right)}{10}$$

### Disease mapping using a biology-based model

In the first step, mapping of environmental risk factors for *P. vivax* malaria, considering temperature and YRH individually, was done using ArcGIS version 9.1 (ESRI; Redlands, CA, USA). Ordinary kriging, using the geostatistic module, was employed to generate smoothed surface maps of the monthly mean temperature, YRH, and number of months suitable for parasite survival. In order to study the spatial variation in the same season over different decades, the spatial extents of the suitable transmission regions at each of two time points for both *N*_*month*_ and YRH were compared.

In a second step, the individual surface maps of the *N*_*month*_ and YRH were overlaid to predict the potential risk areas of *P. vivax* transmission. Historical records indicate that malaria transmission in P.R. China is geographically restricted to areas south of latitude 45°N [3]. In all malaria endemic regions the transmission period exceeds four successive months of the year. In this study, the regions which meet the condition that *N*_*month*_ > 4 and YRH above 60% were the focus areas. The final malaria prediction map for each decade was overlaid by two masked maps, one showing the number of months suitable for parasite survival and the other the length of YRH map in excess of 60%.

## Results

### Predictive malaria risk maps

**Figure 1:**
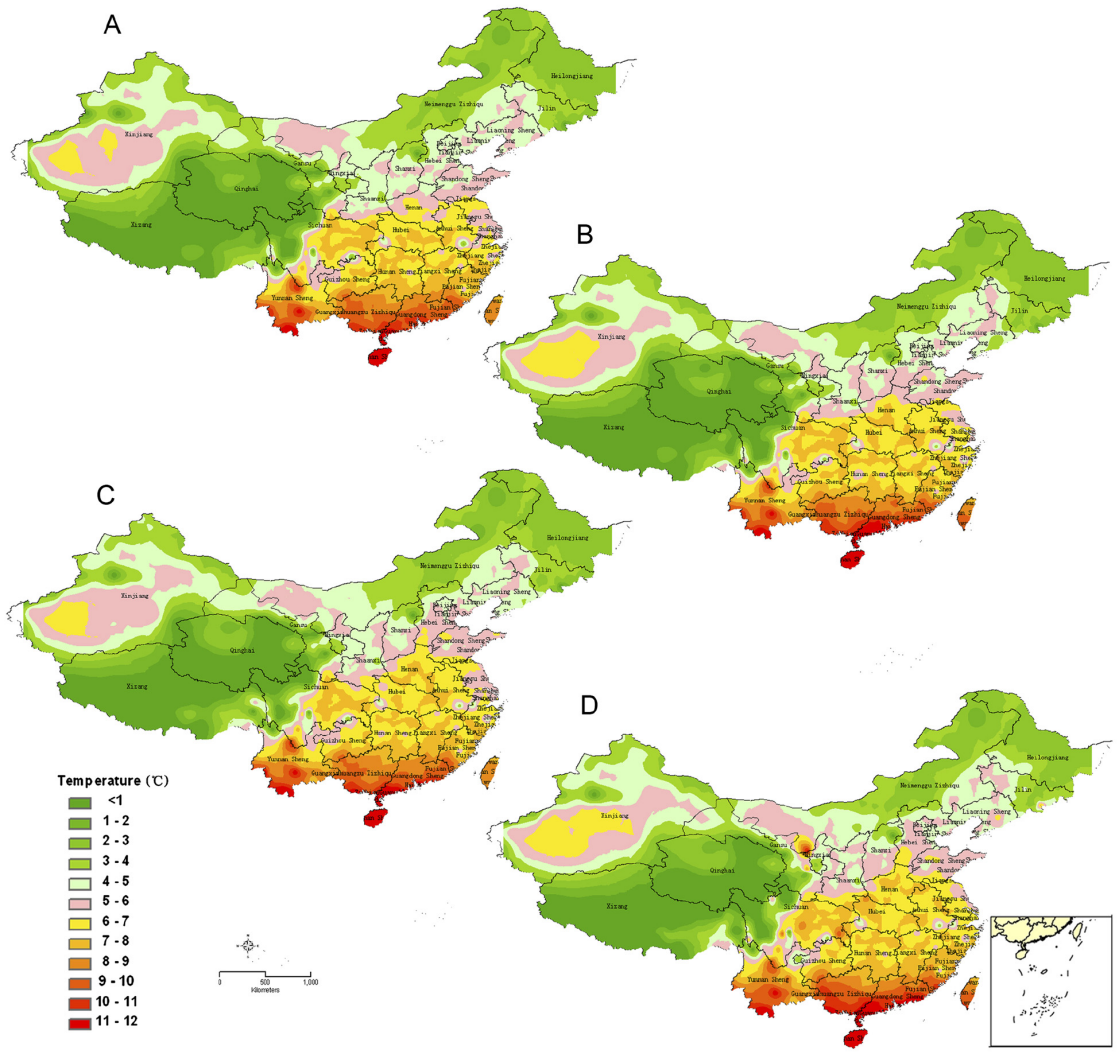
**Prediction maps of malaria transmission showing the number of months in which temperatures are suitable for malaria transmission.** The maps were created based on data derived for 1961–1970 (**A**), for 1971–1980 (**B**), for 1981–1990 (**C**), and for 1991–2000 (**D**), respectively.

**Figure 2:**
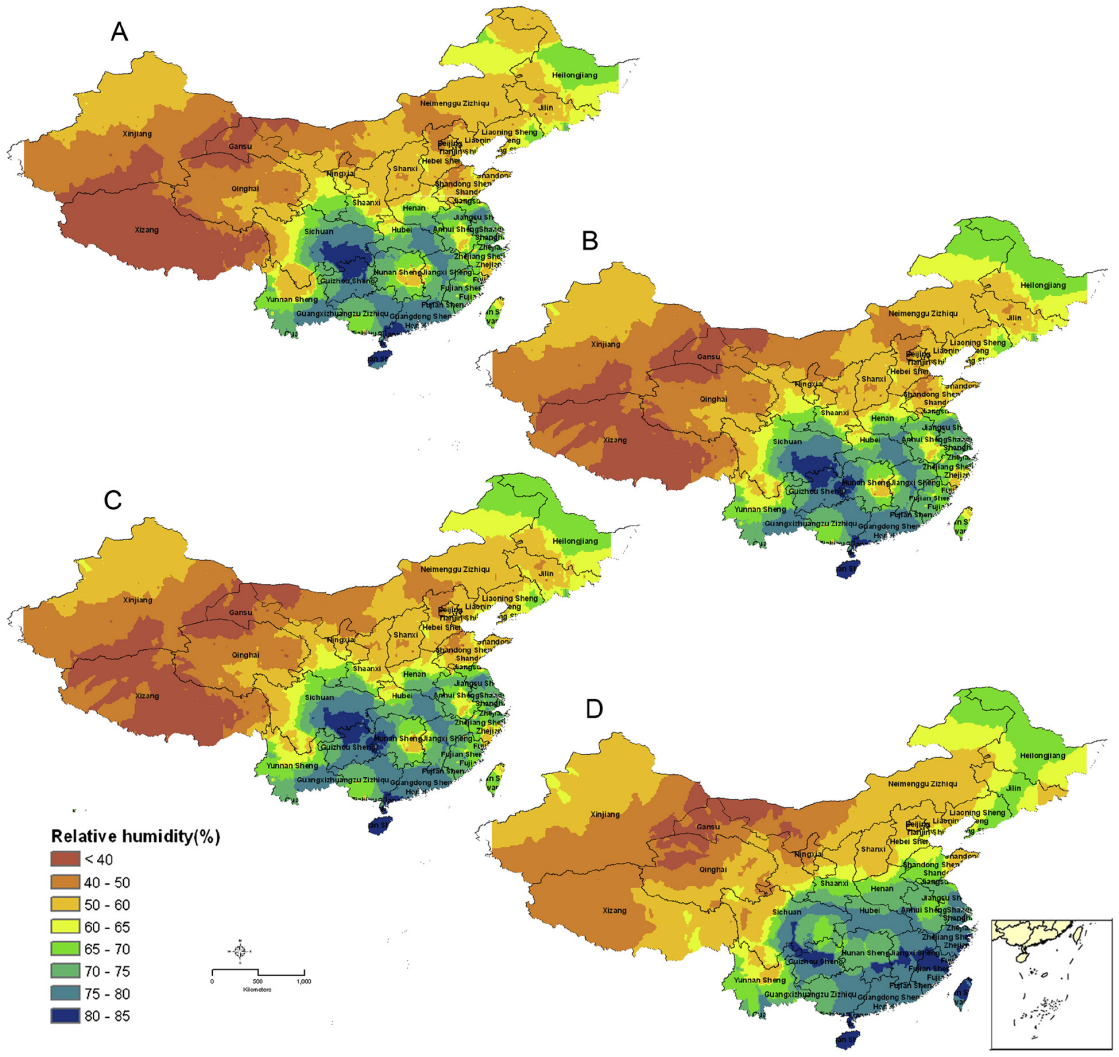
**Prediction maps of malaria transmission in P.R. China based on the relative humidity (%).** The maps were created with data derived for 1961–1970 (**A**), for 1971–1980 (**B**), for 1981–1990 (**C**), and for 1991–2000 (**D**), respectively.

Figures 1 and 2 show predictive *P. vivax* malaria risk maps for P.R. China, based on single environmental determinants. Figure 1 shows that variables suitable for *P. vivax* survival are shifting periodically over the year with respect to the mean temperature, the smallest appropriate areas appearing in January and the largest in July/August. The data suggest that the parasite can survive throughout the year in Hainan and in parts of the Yunnan and Guangdong provinces. South-western plateau areas, such as Tibet and the Qinghai province, are malaria-free throughout the year [18]. Comparing results between different decades, unstable transmission areas (where the transmission period varies between 5 and 6 months) gradually expanded northwards. Many are characterized by prolonged transmission periods, especially in the central part of P.R. China.

The maps based on YRH show a gradual decrease of the RH from coastal areas toward inland. It should be noted that the Tibet plateau has a particular low YRH (Figure 2) due to high altitude coupled with low precipitation and low temperature. Monthly RH values show a cyclical pattern over the years with high RH observed in summer months (July/August) and low RH in winter months. Comparing the results of the YRH over four decades reveals that the northeast of P.R. China is experiencing increasing RH.

**Figure 3:**
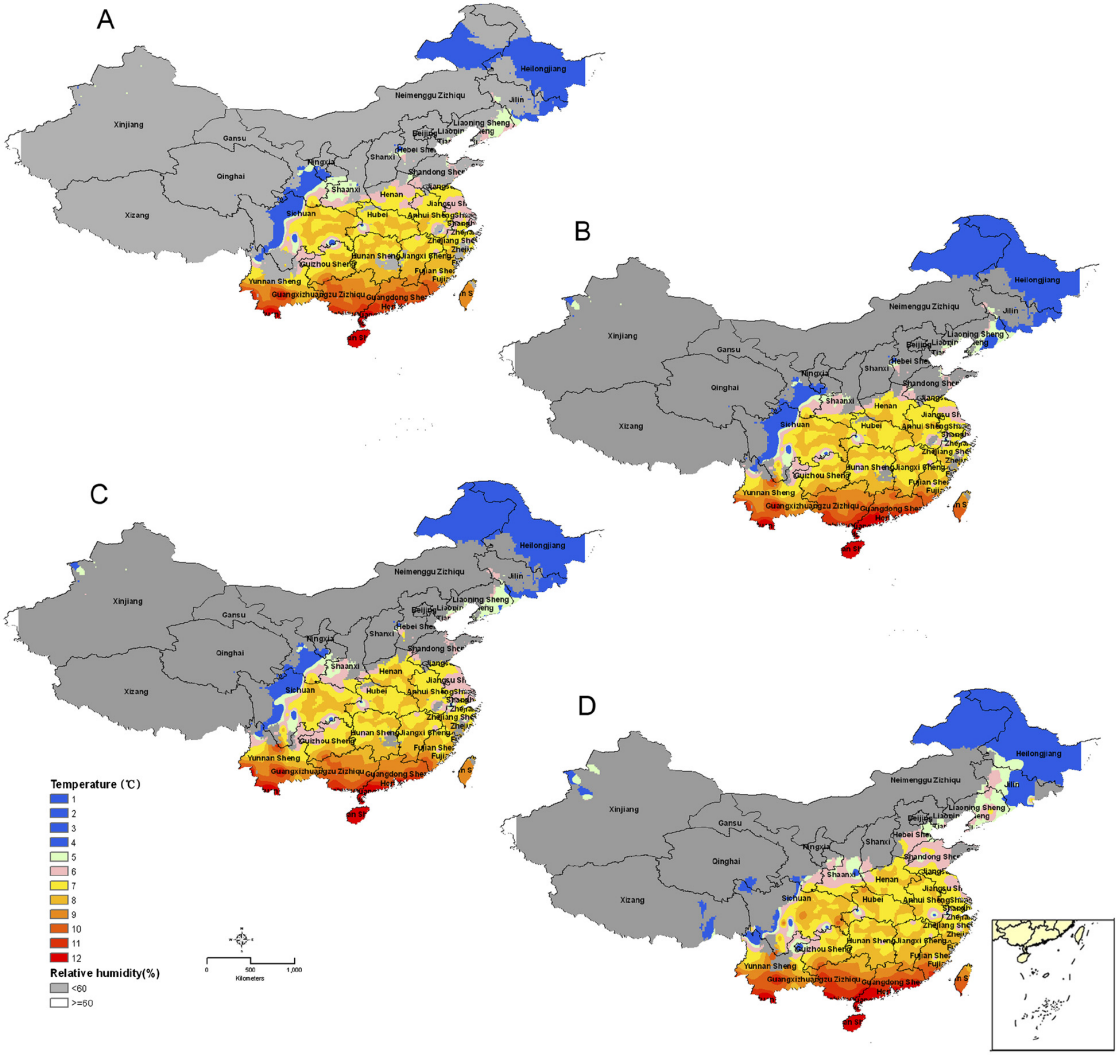
**Prediction maps of malaria transmission in P.R. China by combining two factors: (i) the number of months in which temperature is suitable for malaria transmission, and (ii) relative humidity (%).** Grey colour screens the regions with relative humidity less than 60%. Blue colour indicates the regions where the number of month suitable for malaria transmission is less than 4 successive months. The maps were created based on data derived for 1961–1970 (**A**), for 1971–1980 (**B**), for 1981–1990 (**C**), and for 1991–2000 (**D**), respectively.

Figure 3 shows predictive maps according to multiple environmental factors. These maps predict that *P. vivax* malaria mainly occurs in the south-eastern part of P.R. China with the risk of malaria increasing steadily from north to south. Comparison between different decades shows that there is a high probability that variables suitable for malaria transmission will shift northwards, mainly driven by RH. The areas bordering on the Democratic People’s Republic of Korea (DPRK) emerge as regions potentially suitable for *P. vivax* malaria transmission. The vastness of this region adds to the potential problem.

Table 1 summarizes the estimated number of people at risk in different predicted malaria transmission areas in the face of climate change. These estimates are based on the sixth national census of P.R. China carried out in 2010.

**Table 1:** Risk population in different malaria transmission zones in P.R. China in face of climate change

**Transmission type**	**Provinces**	**No. risk population (1,000)**	**Population density (/km**^**2**^**)**	**Surveillance-response system**
High risk zone	Hainan	7,870	224.86	Both passive and active surveillance approaches with a particular attention to mobile populations
	Yunnan	42,880	108.83	
Climatic sensitive zone	Anhui	59,860	428.80	Passive surveillance in the transmission season from April to November, and active surveillance targetting identified transmission foci
	Henan	92,560	554.25	
	Hubei	60,280	324.26	
	Jiangsu	74,380	724.95	
Potential transmission zone	Jilin	27,280	145.57	Intensified surveillance and responses in border areas
	Liaoning	42,380	290.87	

Note: The risk population is based on the sixth National Census carried out in 2010.

## Discussion

In P.R. China, the transmission and re-emergence of malaria has been restrained through continued efforts, yet malaria remains an important public health problem in the southern and central parts of the country [6]. In 2010, the NMEP was launched with the ultimate goal of interrupting malaria transmission by 2015 in all areas, except the border areas of Yunnan province due to cross-border transmission, and malaria elimination all over P.R. China by 2020 [28]. This ambitious goal entails that the NMEP needs to establish effective surveillance-response systems tailored to local transmission patterns.

Currently, the malaria transmission in P.R. China can be divided into three major strata in terms of intensity [29]. The first stratum is located in the southern and south-western regions, i.e., Yunnan, Hainan, Guangxi and Guangdong provinces, which have experienced transmission, both of *P. falciparum* and *P. vivax*, for a long time [4,5]. Recently, in Hainan an exceedingly low level of *P. falciparum* malaria was reached, though the transmission of *P. vivax* was persistent [11]. In Yunnan, on the other hand, there is still considerable transmission of *P. falciparum*, mainly due to the importation through mobile population in the southern border areas [10]. Guangdong province is experiencing fast economic growth and the malaria incidence rate is reaching very low level [30]. The second stratum focuses on the central part of the country (i.e., Anhui, Henan and Jiangsu provinces), where the transmission of *P. vivax* shows highly unstable patterns [11]. For instance, an outbreak of malaria occurred in Anhui in 2006 [31], and this province has still one of the highest malaria incidences in P.R. China, followed by the provinces of Henan, Hubei, and Jiangsu [11]. The remaining areas, where malaria transmission is very low or might have been interrupted, belong to the third stratum, and hence only few imported cases are reported every year. Of note, imported malaria cases have become a growing challenge for the NMEP, which might be explained by climate change and the lack of effectiveness of the malaria surveillance system. Consequently, there is a pressing need for a more effective surveillance-response strategy that must be tailored to the transmission/importation pattern for the aforementioned strata.

**Figure 4:**
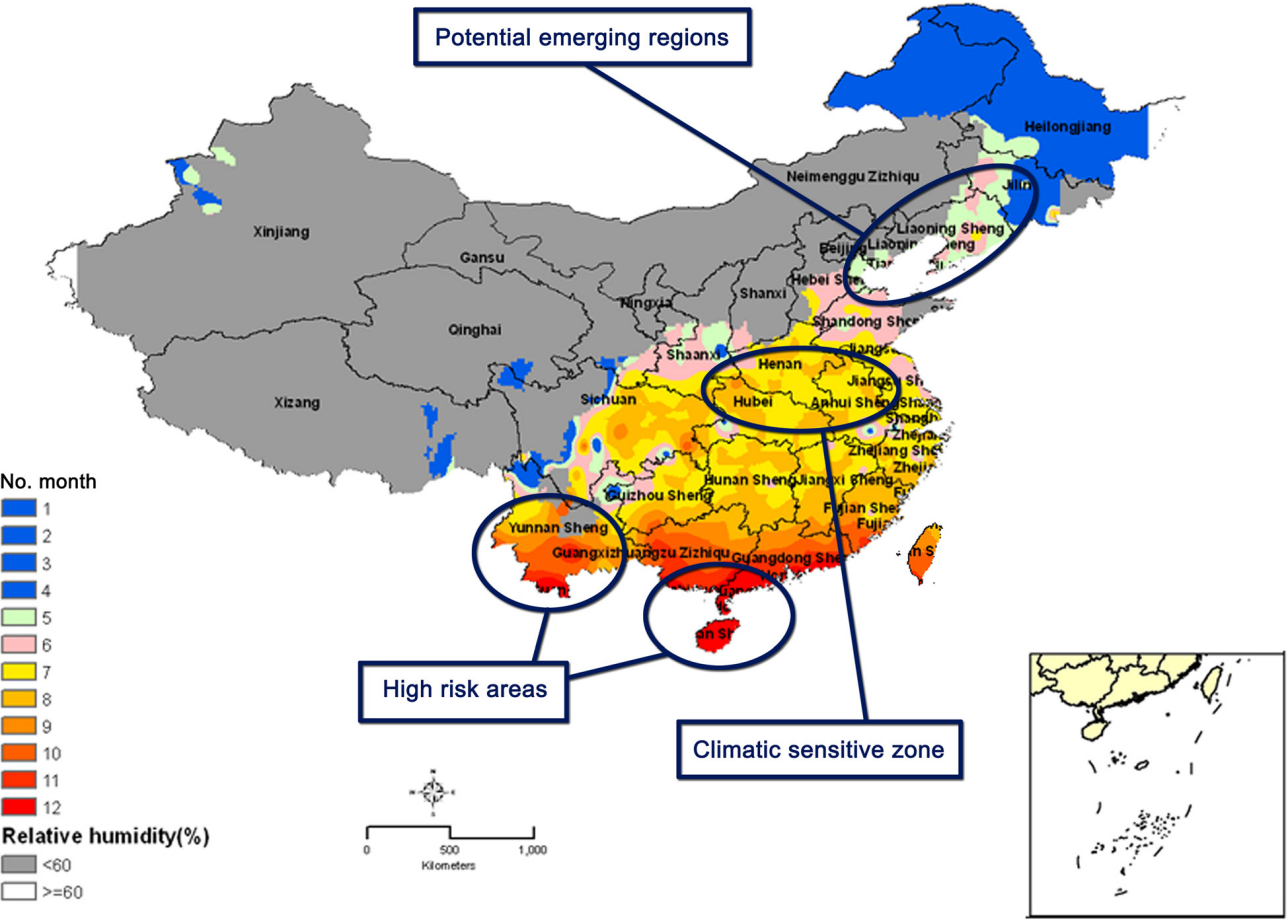
**The maps of malaria transmission highlighting the surveillance-response strategies.** In the southern and south-eastern region where is the high risk areas for malaria transmission, the passive and active surveillance approaches identified. In the central region where is the climate sensitive zone to the malaria transmission, an appropriate surveillance-response approach is necessary to develop. In the north-eastern region where is potential malaria emerging regions due to climate change, the continuous surveillance and monitoring is warranted.

In order to understand the spatial-temporal transmission patterns of each epidemiological region in face of climate change, the current analysis predicts the regions of malaria transmission risk in P.R. China. Our findings highlight the regions that require most attention and surveillance showing in Figure 4. The results show that the projected whole-year malaria transmission areas are still in the southern and south-eastern parts of the country, namely Yunnan, Hainan, Guangxi and Guangdong. While endemic areas of *P. falciparum* have remained restricted to Yunnan and Hainan, *P. vivax* have reported in Yunnan, Hainan, Guangxi and Guangdong provinces, while high transmission mainly occurs in the bordering region of Yunnan and the hilly-forested south of Hainan [11]. Although Hainan has reached at lower level of *P. falciparum* transmission, the passive and active surveillance approaches of the whole population – with particular attention to mobile populations – needs to be strengthened and followed by effective public health response actions when aiming at elimination. Passive surveillance is the routine reporting of the cases via hospital reporting system, while active surveillance is done for finding cases in the community mainly through door-to-door surveys.

When turning to the possible effect of climate change to the unstable malaria transmission regions in the central part of P.R. China (i.e., Henan, Anhui, and some parts of Jiangsu), prolonged transmission periods are found, previously covering the months of May to October but now starting in April. Indeed, two large-scale outbreaks of *P. vivax* malaria occurred in the early 1960s and 1970s with an estimated number of 10 million and 21 million cases, respectively, with the corresponding prevalence figures around 1.5% and 3% [2]. Even now, the number of malaria cases in the Anhui, Henan, Jiangsu, and Hubei provinces account for two-third of the total number of malaria cases in P.R. China, thus higher than that in the hyper-transmission areas of Yunnan and Hainan for five consecutive years. Therefore, it is assumed that Anhui, Henan and Jiangsu, due to climate change, are the most vulnerable provinces with respect to malaria transmission. This in turn implies that the elimination activities for *P. vivax* should focus on the central region, including northern Anhui, eastern Henan, and western Jiangsu. Consequently, NMEP is challenged to develop an appropriate surveillance-response approach that should consist of passive surveillance in the transmission season from April to November and active surveillance concentrated on identified transmission foci.

The predictive *P. vivax* malaria transmission maps indicate that marginal transmission areas have grown, especially since the 1990s. A previous study found that RH is one of the key restriction factors for the distribution of the mosquito vector. No malaria transmission occurs where the yearly average RH is below 60% [14]. Here, it was found that the regions with YRH > 60% have continued to grow over time, particularly in the north-western part of P.R. China (Figure 2). It was reported that local malaria transmission cases in the DPRK, which borders on Jilin and Liaoning, have increased gradually and the transmission period has seemingly become longer [32]. It was suggested that continuous surveillance and monitoring is warranted to prevent further expansion of *P. vivax* malaria caused by climate change involving also this country. This observation makes it important for P.R. China to call for intensified surveillance and responses by NMEP in border areas with DPRK in addition to the ongoing efforts at the southern border of Yunnan province.

In this study, according to model parsimony theory [33], only the most important environmental parameters were utilized to predict malaria transmission at the macro scale. Although multiple environmental factors were considered to predict the potential malaria transmission areas but did not include the determinants linked to societal change, politics, ongoing health interventions, and economics. It has been acknowledged that failing to take these factors into account might overestimate the effects of climate changes on altering the prevalence and incidence of disease [34,35]. It has also been speculated that malaria will extend current geographical distribution, but there is also debate about potential over-emphasizing the effects of climate change [36]. In the future, prediction map at the micro scale, effects of mosquito life-history traits (biting frequency, longevity, population dynamics, vector survival probability [37,38]) should be taken into account. In addition, biological factors, such as distribution and transmission capacity of four main species of *Anopheles* mosquitoes in P.R. China, e.g. *Anopheles sinensis*, *An. anthropophagus*, *An. minimus*, and *An. dirus* vary in different parts of P.R. China. The former and latter factors, in turn, call for more specific prediction maps based on a number of targeted research-action approaches for the different malaria transmission areas experiencing different levels of control or elimination to reach the basis for integrated surveillance-response strategies within NMEP that will hopefully lead to elimination of malaria in P.R. China, currently aimed for by 2020.

## Competing interests

The authors declare that there are no competing interests.

## Authors’ contributions

GJY and ZXN conceived the study and analyzed the data. GJY wrote the first version of the manuscript. XNZ, MT, JU, JBM, RB revised the manuscript. All authors read, contributed to, and approved the final version of the manuscript.
